# Modular Architecture of a Non-Contact Pinch Actuation Micropump

**DOI:** 10.3390/s120912572

**Published:** 2012-09-13

**Authors:** Pei Song Chee, Rashidah Arsat, Tijjani Adam, Uda Hashim, Ruzairi Abdul Rahim, Pei Ling Leow

**Affiliations:** 1 Faculty of Electrical Engineering, Universiti Teknologi Malaysia (UTM), 81310 Johor Bahru, Johor, Malaysia; E-Mails: pschee2@live.utm.my (P.S.C.); rashidah@fke.utm.my (R.A.); ruzairi@fke.utm.my (R.A.R.); 2 Institute of Nanoelectronic Engineering, Universiti Malaysia Perlis (UniMAP), 01000 Kangar, Perlis, Malaysia; E-Mails: tijjaniadam@yahoo.com (T.A.); uda@unimap.edu.my (U.H.)

**Keywords:** electromagnetic micropump, diffuser, lab on chip, hot embossing

## Abstract

This paper demonstrates a modular architecture of a non-contact actuation micropump setup. Rapid hot embossing prototyping was employed in micropump fabrication by using printed circuit board (PCB) as a mold material in polymer casting. Actuator-membrane gap separation was studied, with experimental investigation of three separation distances: 2.0 mm, 2.5 mm and 3.5 mm. To enhance the micropump performance, interaction surface area between plunger and membrane was modeled via finite element analysis (FEA). The micropump was evaluated against two frequency ranges, which comprised a low driving frequency range (0–5 Hz, with 0.5 Hz step increments) and a nominal frequency range (0–80 Hz, with 10 Hz per step increments). The low range frequency features a linear relationship of flow rate with the operating frequency function, while two magnitude peaks were captured in the flow rate and back pressure characteristic in the nominal frequency range. Repeatability and reliability tests conducted suggest the pump performed at a maximum flow rate of 5.78 mL/min at 65 Hz and a backpressure of 1.35 kPa at 60 Hz.

## 1. Introduction

Micropumps have become essential fluidic control modules in self-contained lab on chip (LOC) platforms. They have been shown to provide good solutions in chemical and biological analysis, especially for DNA hybridization applications [1]. Since the piezoelectric driven micropump was described by van Lintel *et al.* [2], several actuation principles and concepts had been extensively researched in the past decade [3–6]. Electromagnetic actuation has merits of low operating voltage, short response time and high energy density [7,8]. Nonetheless, the integration of magnetic coils into membranes involves tedious fabrication setups and can easily cause membrane tears due to Joule heating effects [9].

Many research efforts have been focusing in optimizing the membrane integration performance. Yamahata *et al.* [7] demonstrated a NdFeB composite membrane via two-step molding. Mixtures of 200 μm size magnetic powder and polydimethylsoloxane (PDMS) were polymerized before being magnetized in a magnet charger. Similar efforts were reported by Khoo *et al.* [10] by electroplating soft magnetic material (Permalloy, Ni_80_Fe_20_) onto PDMS to produce membrane type magnetic actuators. The interaction of the external magnetic field and the integrated Permalloy generates torque to displace the membrane.

Apart from soft material composite, Lee *et al.* [11] mounted a NdFeB hard permanent magnet onto a PDMS membrane with UV glue, utilizing a planar copper micro-coil as external actuator and their characteristic experiments showed 7.2 mL/min flow rates at 200 Hz frequency. Zhou *et al.* [12] developed a magnetic encapsulated composite by covering a permanent magnet with two membrane layers. Shen *et al.* [13] and Pan *et al.* [14] reported an external rotational actuator formed by embedding a permanent magnet onto a horizontally laid rotation shaft of a DC minimotor to provide a low power consumption solution. The micropump flow rate can be controlled via the revolutions per minute (rpm) of the motor. Besides the horizontal motor configuration, Du *et al.* [15] implemented magnetic attracted steel balls in a peristaltic micropump driven by a vertically oriented rotating motor. Back pressure of 10 kPa and 5 mL/min flow rate were achieved at 500 rpm. Most of the reported literature utilized actuator bonding onto membranes, causing residual tensile stress when the membrane is in the rest position (non-actuation state). Eventually, the stress gradient along the membrane depth will deteriorate the film mechanical response and reduce its lifespan.

This paper describes the development of a modular setup for a pinch actuation micropump driven by a electromagnetic solenoid, its fabrication and characterization. The solenoid plunger only pinches the membrane during the actuation state and has no contact with the membrane in the rest state, ensuring stress-free conditions. A planar diffuser configuration is used to enable the disposability and rapid prototyping features of this modular micropump, where research on diffuser design optimization is extensively studied [16,17]. Experimental investigations were carried out to determine the influence of separation distance between magnetic plunger and membrane toward micropump flow rate and back pressure performance. Maximum flow rate and back pressure were reported as 5.78 mL/min and 1.35 kPa, respectively, at a separation gap of 2.5 mm.

## 2. Design and Simulation

**Figure 1.**
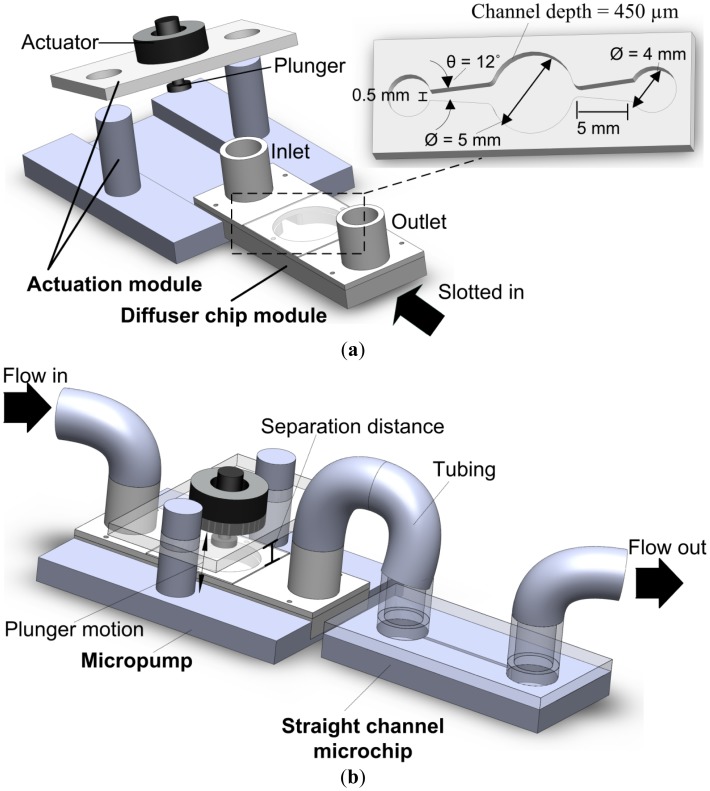
Project Overview. (**a**) Schematic diagram of micropump setup. (**b**) Connection method with the microchip.

Figure 1(a) shows a 3-D schematic illustration of the modular micropump setup and the dimensions of the diffuser design. The connection method between the proposed micropump and a microchip is shown in Figure 1(b).

**Figure 2.**
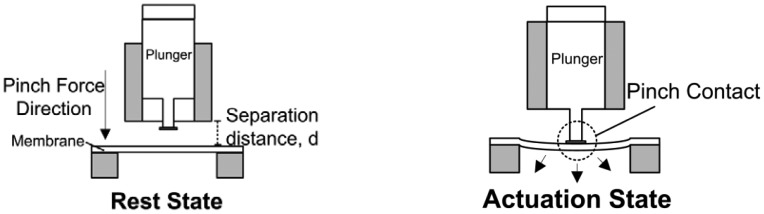
Schematic illustration of pinch actuation operation in rest and actuation mode.

The modular architecture consists of two separate modules: an actuation module and a diffuser chip module. The diffuser chip module can be slotted into the actuation module and perform the micropumping operation. The pump is actuated via a non-contact pinch mechanism. Figure 2 illustrates the non-contact operation of the modular micropump. In rest mode, the magnetic plunger moves upwards. The membrane is naturally flat in tense-free state in the absence of pinch pressure.

Under the actuation mode (pump mode), the downward push of the plunger creates a contraction stroke to expel the fluid through the outlet valve. The continuous cycles between rest and actuation mode create a net fluid flow.

**Figure 3.**
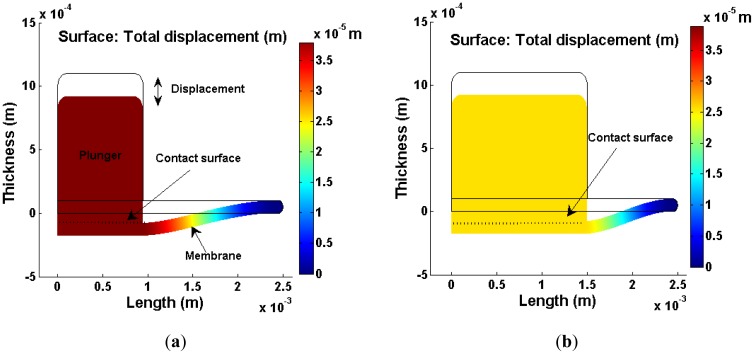
Membrane deflection caused by body load, 100 N/m^3^. (**a**) 3.89 × 10^−5^ m of deflection at 0.38 contact ratio. (**b**) 2.55 × 10^−5^ m of deflection at 0.60 contact ratio.

To equip this driver as a displacement source, the mechanical response towards the surface contact between plunger and membrane was investigated using finite element analysis (FEA). Figure 3(a,b) shows the FEA models of the membrane displacement with different contact surface via COMSOL Multiphysics under 2-D axial symmetry configuration. A mesh sensitivity test was conducted to ensure the analysis result is independent of the meshing densities. The density, Young's modulus and Poisson's ratio of the PDMS material for the membrane are 965 kg·cm^−3^, 750 × 10^3^ Pa and 0.49 [13,18], respectively.

**Figure 4.**
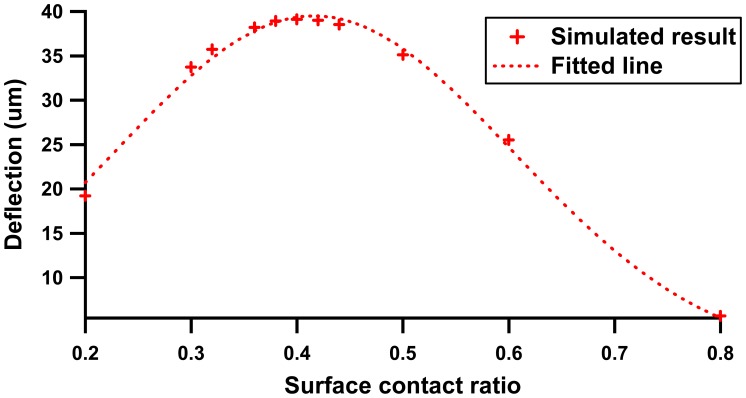
Membrane deflection with surface contact ratio variation.

The simulated result shows the membrane deflection distribution forms a trapezoidal shape, where maximum deflection is concentrated in the pinch region. It is found that a smaller contact ratio (plunger surface to membrane surface) contributes to a larger displacement. Figure 4 shows the membrane deflection profile with respect to the surface contact ratio.

The deflection profile shows a Gaussian characteristic where optimal deflection is promised within surface contact ratio range from 0.3 to 0.45. At a lower contact ratio, the small deflection is mainly attributed to the dominant membrane inertia, whereas beyond a range of 0.45, membrane displacement decays rapidly with the increment of contact ratio. This phenomenon shows good agreement with the equation of centerline displacement, y under subjected applied force, F_d_ per unit cross section area, A_d_ [19]:

(1) $$\frac{\left(\frac{{\text{F}}_{\text{d}}}{{\text{A}}_{\text{d}}}\right){\text{d}}_{\text{m}}^{4}}{16\text{E}{\text{t}}_{\text{m}}^{4}}=\frac{5.33}{\left(1-\nu^{2}\right)}\frac{\text{y}}{{\text{t}}_{\text{m}}}+\frac{2.6}{\left(1-\nu^{2}\right)}{\left(\frac{\text{y}}{{\text{t}}_{\text{m}}}\right)}^{3}$$

where d_m_, t_m_, E and ν represent the membrane diameter, thickness, Young's modulus and Poisson ratio, respectively. Based on the FEA result, a surface contact ratio of 0.4 (2 mm of plunger surface area to the 5 mm membrane diameter) was used in the experimental configuration.

## 3. Microfabrication Technology

For the fabrication of the device, we used poly(methylmethacrylate) (PMMA) thermoplastic. PMMA shows good optical properties as it is transparent, easy to fabricate at low temperature and economical. The developed micropump was composed of two PMMA layers (each with thickness of 2 mm). The bottom layer consists of the imprinted diffuser channel and the top layer consists of the drilled holes for fittings and the chamber. The PMMA microstructure patterning of the bottom layer utilized hot embossing techniques, which has had its rapid prototyping potential demonstrated.

**Figure 5.**
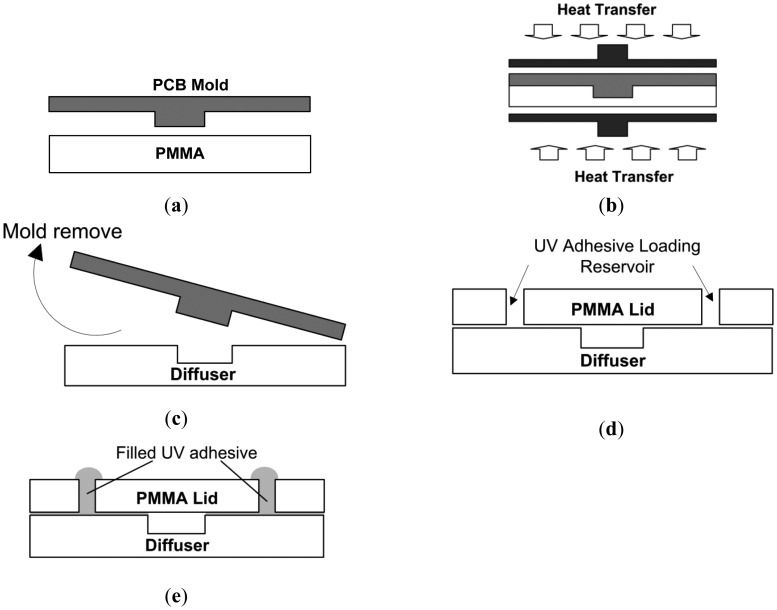
Fabrication procedure of micropump realization. (**a**) Subtract aligned configuration. (**b**) Clamp heat transfer. (**c**) Mold removed. (**d**) Lid assembly with UV interstitial bonding. (**e**) Complete micropump.

Printed circuit board (PCB) technology is well suited for fluidic system realization and this had been reported in other studies [20,21]. Nonetheless, most of the reported literature that utilized PCB substrates as pump body components, such as pump chambers and diffuser conduits has limited applications in the biomedical field due to its bio-compatibility issues. In this work, a 450 μm bulged PCB layer (engraved deep into the copper and FR4 layer) was utilized as mold master and a PMMA subtract was used as the replica component. As compared to photoresist and silicon wafer templates, PCB works as an alternative option for mold template selection which in addition, promises rigid and fast thermal response characteristics. The PCB mold was fabricated via a Circuit Board Plotter (LPKF Proto Mat95S/11) and Figure 5 illustrates the PMMA micropump fabrication protocol and bonding procedure.

Firstly, a PMMA sheet with geometry chip size, 32 mm × 15 mm (length × width) was carefully aligned with the PCB mold master template (shown in Figure 5(a)). Next, the configuration was sandwiched within two aluminum plates and was heated in a preheated oven at 120 °C for 20 minutes. Similar to previous work [22], uniform pressure was exerted on the whole assembly via a G-clamp spindle, as depicted in Figure 5(b). The exerted force was monitored by clamp turn angle, indicated with a 360° protector which was originally attached to the turn spindle. The clamp was periodically tightened up to ensure conformal contact between mold and PMMA substrate. At the PMMA transition temperature, 106 °C, PMMA polymer substrates will melt and gradually fill the mold cavity, forming the diffuser channel. Finally, the PMMA substrate was removed from the oven and de-molded immediately to avoid device cracking due to internal stress (Figure 5(c)). Figure 5(d,e) shows the adhesion of the PMMA cover lid (with predrilled chamber and inlet and outlet reservoirs) to the diffuser layer via the UV interstitial technique under room temperature [23]. Further elaboration of the bonding procedure is discussed in the literature [24].

**Figure 6.**
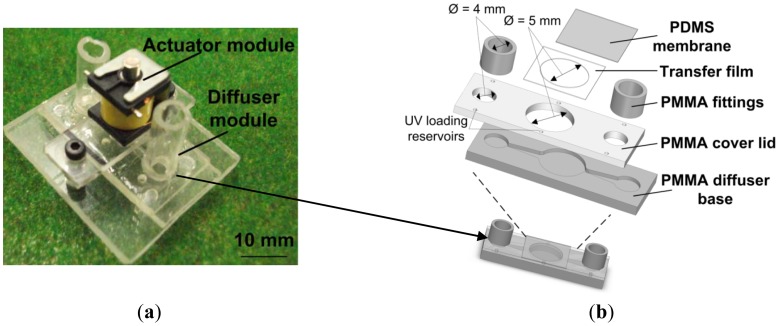
Photograph of the final product. (**a**) Fully assembled micropump with complete module. (**b**) Burst view of the diffuser module with assembled layer.

The stroke volume produced by the actuator was highly dependent on the membrane compliance, where a large Poisson ratio is mandatory to ensure optimal deflection. PDMS Sylgard 184 (Dow Corning Corp, Midland, MI, USA) was prepared at mixing ratio 10:1 and spin coated at 1,000 rpm for 20 seconds on a flat substrate. The PDMS membrane was cured in the oven at 80 °C for 30 minutes. The 100 μm thick flat PDMS sheet was cut into smaller sheet of 20 mm × 15 mm and adhered onto the PMMA lid by using ARclad^®^ IS-8026 (Adhesive Research Inc., Glen Rock, PA, USA) silicon transfer film technology. Figure 6(a) shows the end product of the full micropump assembles with dimensions of 32 mm × 25 mm × 15 mm. The assembled layer of diffuser module is shown in Figure 6(b).

To prolong membrane lifespan, thermal bonding techniques are avoided for both PMMA-PMMA and PMMA-PDMS bonding to eliminate thermal stress that can affect the bonding surface.

## 4. Experimental Setups

### 4.1. Setup for Solenoid Actuator Analysis

The C-frame push type miniature solenoid (Series 45B, BLP, UK) was modified by placing a NdFeB/N40 permanent magnet (diameter, Ø = 3 mm, height = 3 mm) on the iron plunger, creating a latch actuator. The plunger pushed or extended to the end and pulled back to its original position with respect to the induced electromagnetic field applied. To evaluate the inductive characteristics of the modified solenoid, determination of current profile under unload condition is essential for optimizing the pump configuration. A 33 Ω shunt resistor was connected in series to form an RL circuit configuration. A digital multimeter (Tenma Test Equipment, Model 72-7732) was probed across a resistor to provide real time current information and impedance behavior of the actuator. The data was then further analyzed and processed in the MATLAB interface. A function generator (GW Instek, GFG 8210) was employed to drive the solenoid with square wave frequency sweep in the range of 5–80 Hz.

Another indicator in actuator performance evaluation is the gap separation study (*cf.* Figure 2) between the lower side of the plunger and membrane. The pinch force of the solenoid was measured by placing the pump on a digital scale (Shimadzu, Tx 323L) with the solenoid actuator mounted on a digital caliper. The caliper reading indicates the membrane gap separation distance. Five weight measurements were taken for two operating currents, 0.06 A and 0.07 A, to investigate the plunger current behavior with respect to the membrane.

### 4.2. Setup for Characterization of Micropump Performance

The actuator of the micropump was powered up using a square wave voltage supply, V_pp_ 19.6 V of 50% duty cycle where the ambient temperature recorded was 19.5 °C. De-ionized (DI) water worked as the flowing fluid within the channel for all the experiments in this paper. The flow performance of the micropump was evaluated based on frequency variation of the actuator. The pump performance was investigated in two operating frequency ranges, 0–5 Hz (small step increments of 0.5 Hz, to test flow rectification in a lower actuation frequency cycle) and 0–80 Hz (with 10 Hz increments per step). The choice of flow rate was arbitrary selected and was within the range (<1 kHz) reported by Farid *et al.* [25] under electromagnetic actuation. The flow rate-frequency dependence measurement was conducted at zero back pressure, based on mass discharge density at each actuation frequency. The micropump hydrostatic backpressure was measured by taking liquid level difference between the inlet and outlet tubing.

## 5. Results and Discussion

### 5.1. Solenoid Actuator Impedance Analysis

**Figure 7.**
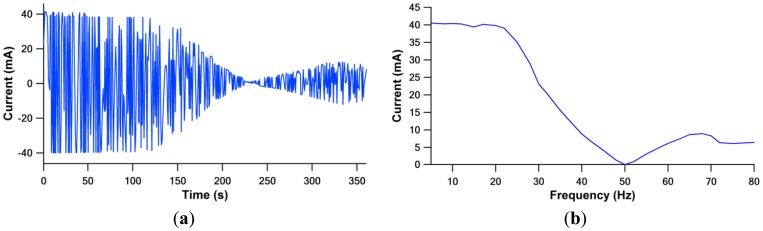
Actuator characterization. (**a**) Impedance profile with time domain variation. (**b**) Impedance study in frequency domain.

The solenoid static operation as a function of frequency was plotted in the time domain and frequency domain, as depicted in Figure 7. The current plot profile represents solely actuator behavior in the absence of fluid structure interaction of the pump and its hydraulic pressure performance.

From Figure 7, the current signal acquired shows optimum profile at lower frequency range. This can be monitored from the impedance, Z, equation:

(2) $$Z=\sqrt{R^{2}+{\left(2\pi \text{fL}\right)}^{2}}$$

where R, f and L represent resistor, the operating frequency and inductor, respectively.

At the lower frequency range of 5–20 Hz, the acquired signal shows maximum current consumed at minimum impedance. The impedance value was dominated by resistance, due to the low inductive reactance at low frequency. The solenoid had reached its resonant frequency. As the frequency increases, the inductance reactance increases and results in lower current consumption. Another current magnitude peak is observed at the frequency of 65 Hz, due to the phase angle changing between voltage and current.

### 5.2. Electromagnetic Pinch with the Function of Gap Separation Analysis

**Figure 8.**
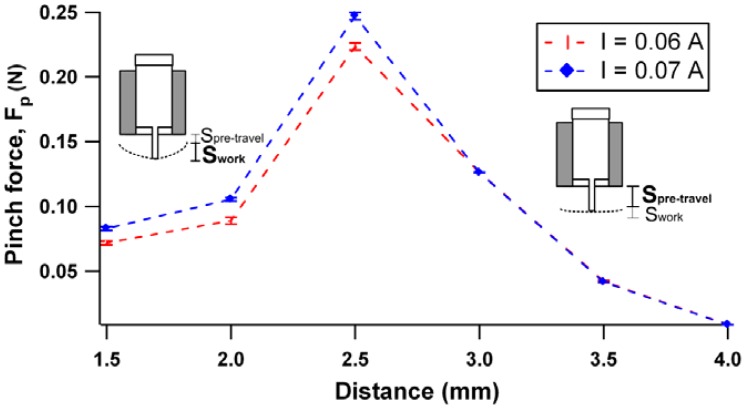
Pinch force exerted on the membrane with gap separation variation.

The pinch force behavior for two current range, 0.06 A and 0.07 A is shown in Figure 8 at different separation distance between the plunger and the membrane.

The obtained result illustrates that the maximum pinch force can be achieved at an optimum gap separation of 2.5 mm. The force starts to deteriorate with further increases of the separation gap. This phenomenon can be related to the plunger stroke mechanism, where the total stroke is composed of two components: pre-travel stroke and working stroke. The former is known as the plunger movement before the pickup of membrane load. At greater pre-travel stroke, the major contributed to the total force is from the plunger momentum force rather than the electromagnetic force.

This scenario can be observed from Figure 8 where the force produced is independent of the current supply when the separation distance was further increased to 3 mm. Working stroke takes place in the 1.5 mm–3.0 mm region, where the force is against membrane elastic compliance. In this state, large current supplied leads to larger induced electromagnetic force, thus, dominating the total produced force. Subsequently, three gap separation configurations: 2.0 mm, 2.5 mm and 3.5 mm with current value of 0.07 A will be implemented for experimental characterization.

### 5.3. Characterization of Micropump Performance

**Figure 9.**
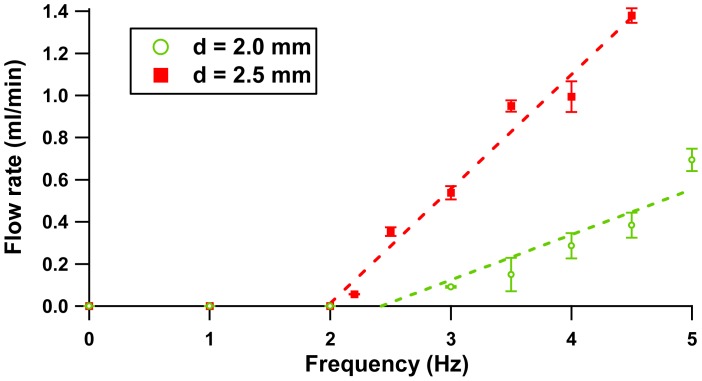
Flow rate *vs.* Frequency (0–5 Hz).

The micropump flow rate response was studied experimentally at various actuation frequencies. Figure 9 depicts the flow rate at low actuation frequency with 1 Hz resolution.

**Figure 10.**
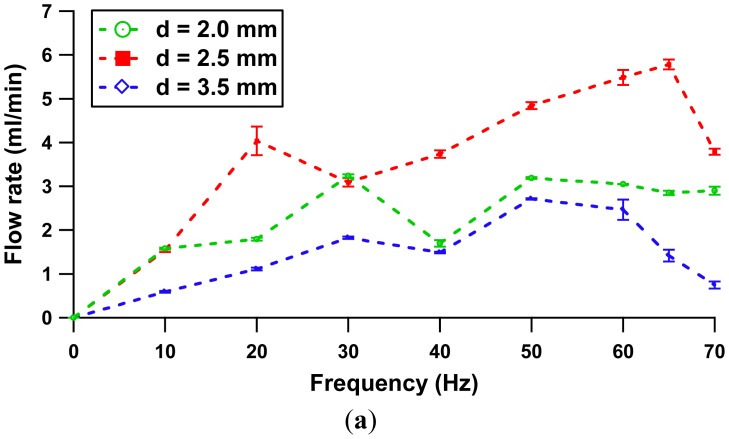

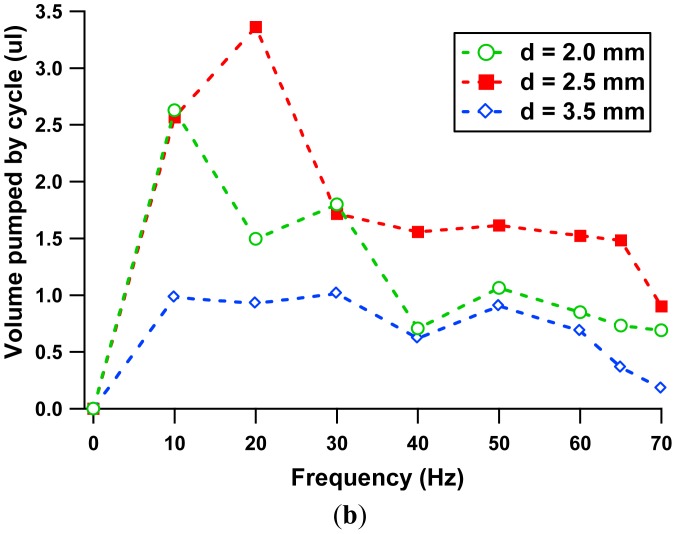
(**a**) Flow rate *vs*. Frequency (0–70 Hz). (**b**) Volume pumped by cycle *vs.* Frequency.

Figure 9 illustrates how the flow rates for separation gap distances of 2.0 mm and 2.5 mm increase linearly with respect to the driving frequency applied. The error plot shows the experimental error is less than 10% of its average value. From the experiment, the flow rectification is achievable at a minimum flow rate of 56.7 μL/min at a frequency of 2.2 Hz (separation of 2.5 mm) and 91.2 μL/min at a frequency of 3 Hz (separation of 2.0 mm). Nonetheless, the configuration with separation distance of 3.5 mm does not show any rectification response at this actuation range due to the fact the low force produced is not sufficient to overcome the fluid inertia term. Figure 10(a) shows flow rate properties at a nominal frequency range of 0–70 Hz. Further characterization of volume pumped by cycle is included in Figure 10(b).

A dip at a driving frequency of 20 Hz was observed at 2.5 mm gap configuration in Figure 10(a). This phenomenon can be related to the current consumption characteristic by the actuator (refer to Figure 7), as the actuation frequency is close to natural frequency of the actuator, the membrane vibrates at optimal amplitude, producing a high flow rate. The system had reached its actuator resonance. A further increase to 30 Hz of frequency will lead to a rapid decrease of flow rate and reaches a local minimum. Beyond 30 Hz, the flow rate increased with the function of frequency. A second dominant peak flow rate was noted at the frequency of 65 Hz. The second peak is noticed to be higher compared to the first peak.

Briefly, the pump performance is dictated by two flow velocities: volumetric oscillation flow (strongly dependent on the solenoid characteristic) and net flow rate (dependent on diffuser channel design). The former term refer to the flow inside the pump chamber, whereas the latter illustrates the difference in velocities between forward and backward flow [26]. The statement is more vividly explained with Figure 10(b). The plot shows the maximum volume pumped by each cycle (maximum volumetric oscillation flow) occurred at 20 Hz, which explains the existence of the first peak. As the flow rate is the product of stroke volume and operating frequency, an increase in operation frequency under constant stoke volume will lead to the formation of the second flow rate peak (5.78 mL/min) at 65 Hz. Hence, this characteristic suggests that the system has reached its pump resonance. Beyond 65 Hz, the net flow rate decays as there are no appreciable fluid impedance differences between the inlet and outlet conduits.

**Figure 11.**
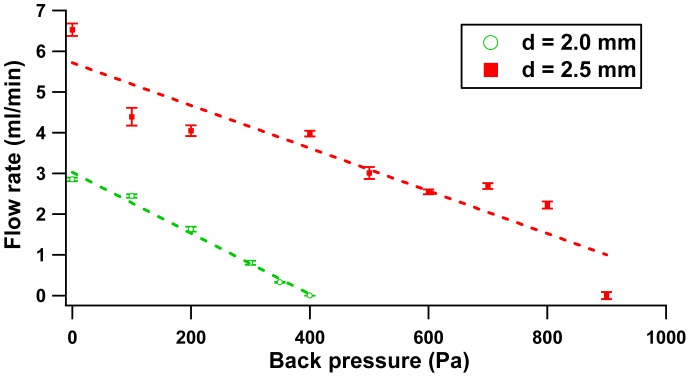
Flow rate *vs.* Back pressure.

On the other hand, the configurations with separation of 2.0 mm and 3.5 mm exhibit same flow patterns. Two peaks were featured at the frequency of 30 Hz and 50 Hz and a local minimum occurred at 40 Hz. In this case, the substantial net flow rate has more effect on the peak displacement than the trivial volumetric oscillation flow contributed by the pinch force. The pinch force effect (proportional to volumetric oscillation flow) is only prominent at an optimum distance of 2.5 mm. The flow rate-back pressure dependence result at 65 Hz is illustrated in Figure 11.

**Figure 12.**
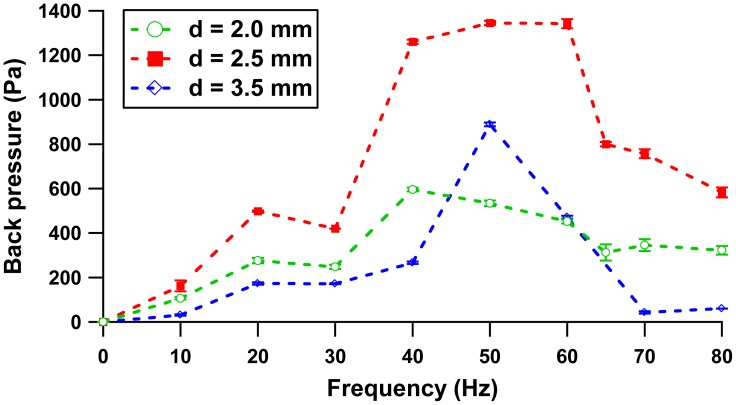
Back pressure *vs.* Frequency.

Figure 11 illustrates the correlation between flow rate and back pressure of 2.5 mm and 2.0 mm distance configuration. The plot follows a linear regression which obeys the behavior of the reciprocating pump, where maximum back pressure occurs at zero flow rate and *vice versa*. Like in Figure 9, the configuration of 3.5 mm gap was excluded in the plot because of the low flow rate produced. Figure 12 reveals the capability of the micropump to oppose free moving flow in the fluidic system.

**Figure 13.**
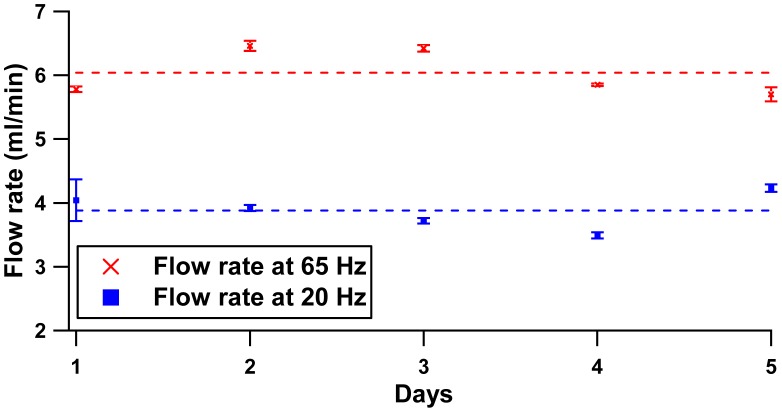
Repeatability and reproducibility Test.

Figure 12 shows that three configurations produced similar trends from 0–40 Hz, where the first peak of the back pressure for all configurations occurs at 20 Hz, while the local minimum is at 30 Hz. In this regime (0–40 Hz), the pressure head is determined by the compression ratio (ratio of stroke volume to the dead volume) [19] and limited by solenoid performance. Beyond 40 Hz, the high operating frequency results in small plunger strokes due to fast cutting of the electromagnetic flux. The small pinch pressure on the membrane contributes to different peaks for each distance separation configuration. The peak was noted at a driving frequency of 40 Hz with 0.6 kPa, 50 Hz with 0.88 kPa and 60 Hz with 1.35 kPa at separation gaps of 2.0 mm, 3.5 mm and 2.5 mm, respectively. Repeatability sets of five flow rate measurements for 5 days are presented in Figure 13. Two peak frequencies (20 Hz and 65 Hz) at the separation distance of 2.0 mm were included in the study plot. Graphical plot of mean and standard deviation of each set were included to describe within test (repeatability) and between tests (reproducibility) measurement.

The small range of flow rate deviation (error bars) for the flow rate of 65 Hz and 20 Hz shows the high repeatability of the test measurements. Similar height patterns between tests across 5 days indicate that the pump is highly reproducible, where 92.72% and 93.95% is reported at 20 Hz and 65 Hz, respectively.

**Figure 14.**
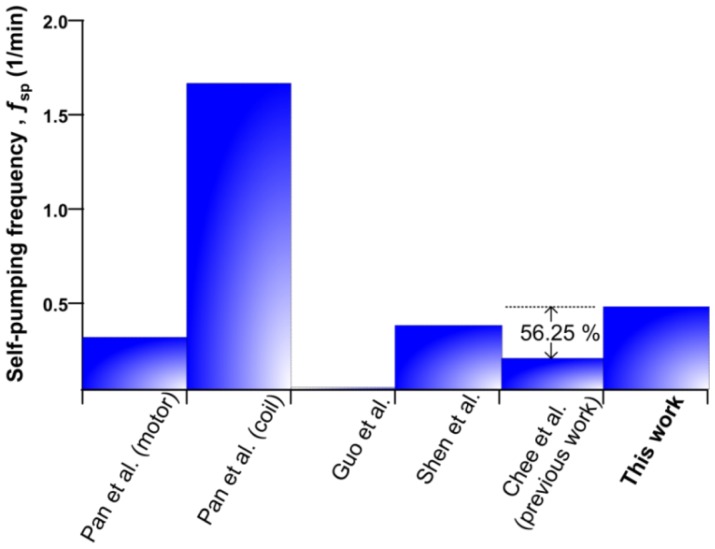
Performance comparison to other reported literature.

As maximum volumetric flow rate is a major key feature in micropump application selection, it is instructive to compare the performance of the electromagnetic micropump with its counterpart reported in the literature. Figure 14 encapsulates the performance of reported micropump based on the ratio of maximum flow rate (mL/min) to package size (mm^3^), which also refer as self-pumping frequency, *f*_sp_ [19]

Pan *et al.* [14] investigated an external micro-motor driven and planar coil micropump. The motor driven scheme provides a good solution for low power applicationa with 0.8 mL/min of flow rate and 2,500 mm^3^ package size. However, the compact planar micro-coil driving micropump scored well in self-pumping frequency due to its relatively small size construction, 600 mm^3^, which can be integrated within a LOC platform as the total size of the device is a major consideration. A similar planar configuration was deployed by Shen *et al.* [27]. More coil turns in a planar actuator with package size 11848 mm^3^ resulted in low self-pumping frequency. The pump performance must be judged by its high flow rate (6.8 mL/min) and back pressure (37 kPa) characteristics. For low flow rate applications, Guo *et al.* [28] proposed a solenoid actuated micropump with a range of 0.05–0.9 mL/min. From Figure 14, the current micropump setup shows 56.25% higher self-pumping frequency ability as compared to our previous pump setup [29]. Apart from the ease of the fabrication method, this micropump offers separate modules where the actuation module can be reused and the diffuser chip module can be replaced.

## 6. Conclusions

A modular micropump architecture driven by a continuous pinch cycle of rest and actuation modes was demonstrated. The micropump was realized by a rapid hot embossing technique, utilizing PCB material as mold template and PMMA polymer as replica product. As a gap separation exists between the plunger and the membrane, therefore, the distance of the gap separation has been evaluated. Gap distances of 2.0 mm, 2.5 mm and 3.5 mm were tested. From the results obtained, a gap with 2.5 mm was associated with highest pinch force (0. 25 N at current of 0.07 A), flow rate (5.78 mL/min) and back pressure (1.35 kPa). To enhance the pump performance, FEA was deployed to investigate the effect of contact surface between plunger area surface and membrane area surface on membrane deflection. The simulated results show that a high deflection of the membrane can be achieved at a surface contact ratio of 0.4. This paper introduces an alternative way to integrate a micropump into a disposable LOC system, where the range of flow rates and back pressures can be adjusted by manipulating the driving frequency of the actuator and adjusting the gap separation between the plunger and the membrane.
